# Near-UV Light Induced ROS Production Initiates Spatial Ca^2+^ Spiking to Fire NFATc3 Translocation

**DOI:** 10.3390/ijms22158189

**Published:** 2021-07-30

**Authors:** Furkan E. Oflaz, Zhanat Koshenov, Martin Hirtl, Rene Rost, Olaf A. Bachkoenig, Benjamin Gottschalk, Corina T. Madreiter-Sokolowski, Roland Malli, Wolfgang F. Graier

**Affiliations:** 1 Molecular Biology and Biochemistry, Gottfried Schatz Research Center, Medical University of Graz, Neue Stiftingtalstraße 6/6, 8010 Graz, Austria; furkan.oflaz@medunigraz.at (F.E.O.); zhanat.koshenov@medunigraz.at (Z.K.); martin.hirtl@medunigraz.at (M.H.); rene.rost@medunigraz.at (R.R.); olaf.bachkoenig@medunigraz.at (O.A.B.); benjamin.gottschalk@medunigraz.at (B.G.); corina.madreiter@medunigraz.at (C.T.M.-S.); roland.malli@medunigraz.at (R.M.); 2 BioTechMed Graz, Mozartgasse 12/II, 8010 Graz, Austria

**Keywords:** UV light, ROS, L-type Ca^2+^ channel, transcription factor, NFATc3 translocation, mitochondrial Ca^2+^ buffering

## Abstract

Ca^2+^-dependent gene regulation controls several functions to determine the fate of the cells. Proteins of the nuclear factor of activated T-cells (NFAT) family are Ca^2+^ sensitive transcription factors that control the cell growth, proliferation and insulin secretion in β-cells. Translocation of NFAT proteins to the nucleus occurs in a sequence of events that starts with activating calmodulin-dependent phosphatase calcineurin in a Ca^2+^-dependent manner, which dephosphorylates the NFAT proteins and leads to their translocation to the nucleus. Here, we examined the role of IP_3_-generating agonists and near-UV light in the induction of NFATc3 migration to the nucleus in the pancreatic β-cell line INS-1. Our results show that IP_3_ generation yields cytosolic Ca^2+^ rise and NFATc3 translocation. Moreover, near-UV light exposure generates reactive oxygen species (ROS), resulting in cytosolic Ca^2+^ spiking via the L-type Ca^2+^ channel and triggers NFATc3 translocation to the nucleus. Using the mitochondria as a Ca^2+^ buffering tool, we showed that ROS-induced cytosolic Ca^2+^ spiking, not the ROS themselves, was the triggering mechanism of nuclear import of NFATc3. Collectively, this study reveals the mechanism of near-UV light induced NFATc3 migration.

## 1. Introduction

As ubiquitous messengers, calcium ions (Ca^2+^) control the fate of the cells by regulating diverse Ca^2+^-dependent pathways and transcription factors, including the nuclear factor of activated T-cells (NFAT) [1]. Among the five members of these transcription factors, four (NFATc1-c4) are dynamically activated by the rise of cytoplasmic Ca^2+^ [2]. Under basal cytosolic Ca^2+^ levels, NFAT proteins are heavily phosphorylated and trapped in the cytosol by masking the nuclear localization sequence (NLS) [1]. However, upon intracellular Ca^2+^ rise, the calmodulin-dependent phosphatase calcineurin is activated and dephosphorylates these transcription factors, exposing the NLS and inducing nuclear translocation [1,2]. Inside the nucleus, NFAT proteins initiate the transcription of several genes and, thereby, control the cell functioning in a Ca^2+^-dependent manner. On the contrary, once the intracellular Ca^2+^ concentration returns to the basal levels, re-phosphorylation of NFAT proteins by nuclear resident protein kinases leads to NFATs’ return into the cytosol by exposing the nuclear export sequence (NES) [3].

Among other tissues, NFAT proteins are expressed in pancreatic β-cells [4] and control the transcription of genes responsible for the proliferation and modulation of insulin secretion [5,6]. Notably, the source of Ca^2+^ ions that drive NFAT proteins’ translocation to the nucleus differs among different cell types. In excitable cells, NFAT proteins are mainly activated via transmembrane Ca^2+^ fluxes through the CaV1 family of L-type voltage-gated Ca^2+^ channels [7,8]. However, activation of NFAT proteins via the L-type Ca^2+^ channel requires a minimum threshold frequency of Ca^2+^ oscillations [9,10]. Interestingly, not all members of the NFAT family are activated by the same frequency of Ca^2+^ oscillations through the L-type Ca^2+^ channel. For instance, only NFATc2 and NFATc3 proteins were shown to be activated upon glucose-induced Ca^2+^ oscillations via L-type Ca^2+^ channel in β-cells, indicating the isoform-specific sensitivity for Ca^2+^ oscillations [10]. 

It is reported that reactive oxygen species (ROS) production also induces NFAT translocation to the nucleus in various cell types [11,12]. Although the source of ROS-induced Ca^2+^ oscillations differs among different cell lines [13,14,15], the effect of ROS production on cytosolic Ca^2+^ oscillations has been reported in multiple cell types [13,14]. In mast cells, it was reported that ROS production leads to cytosolic Ca^2+^ oscillations via the phospholipase C (PLC)/inositol 1,4,5-trisphosphate (IP_3_) pathway [14]. In smooth muscle cells, Ca^2+^ oscillations are generated through the L-type Ca^2+^ channel [13]. While exploring the effect of IP_3_ generating agonist stimulation on NFATc3 translocation, we saw a strong induction of cytosolic Ca^2+^ spiking and NFATc3 translocation by the near-UV light illumination usually applied for measuring cytosolic Ca^2+^ with the most frequently used chemical Ca^2+^ dye, Fura-2. Live-cell imaging revealed that near-UV light triggered ROS production, induces cytosolic Ca^2+^ spiking via the L-type Ca^2+^ channel and yielding NFATc3 translocation to the nucleus. Moreover, we present herein that the mitochondria’s ability to buffer cytosolic Ca^2+^ makes them key regulators in controlling UV light-induced Ca^2+^ spikes and NFATc3 translocation.

## 2. Results

### 2.1. IP_3_ Generating Agonist Triggers Bi-Phasic GFP-NFATc3 Translocation into the Nucleus

**Figure 1:**
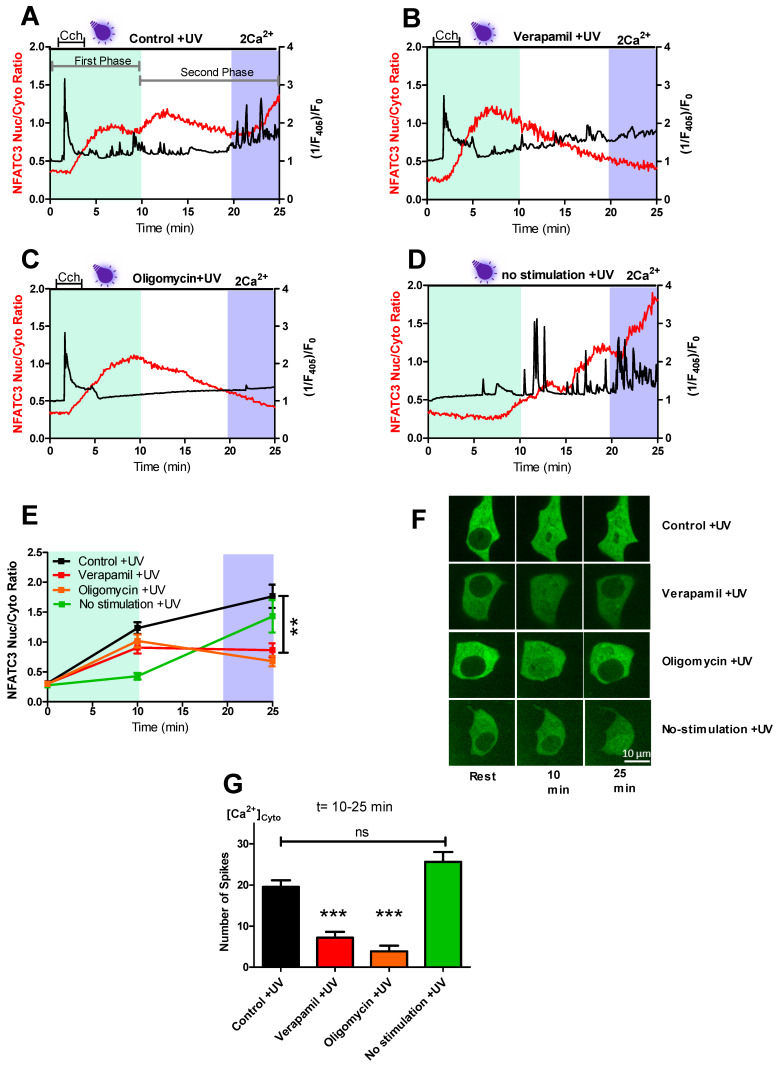
Bi-phasic regulation of nuclear GFP-NFATc3 translocation in INS-1 cells. Representative trace of CCh-induced Ca^2+^ rise (black trace) measured with Fura-2 by exciting the cells with 405 nm laser line. Simultaneous GFP-NFATc3 translocation (red trace) was assessed by exciting the cells with 488 nm laser line in the cells perfused with 2 mM extracellular Ca^2+^ solution in control +UV (**A**), 10 µM verapamil +UV (**B**), 2 µM oligomycin +UV (**C**), and no-stimulation +UV (**D**). Light green color indicates the first phase while white and light blue color indicates the second phase. Time courses of GFP-NFATc3 translocation as MEAN + SEM in control +UV (black), verapamil +UV (red), oligomycin +UV (orange), and no-stimulation +UV (green) assessed by the ratio of nucleus to the cytosol, where min 0 shows the resting ratio of GFP-NFATc3, min 10 shows the maximum translocation ratio of GFP-NFATc3 in the 0 to 10 min time interval, whereas min 25 indicates the maximum translocation ratio of GFP-NFATc3 in the 20 to 25 min time interval (**E**). Representative images indicate the translocation of GFP-NFATc3 in INS-1 cells. The inserted white bar indicates 10 µm (**F**). Bar graphs represent the occurrence of cytosolic Ca^2+^ spiking calculated in the time point between 10 and 25 min (**G**). Significant differences were assessed using one-way ANOVA with Tukey’s multiple comparison test and presented as specific p-values (** *p* < 0.01, *** *p* < 0.001, n.s.—not significant). Control (*n* = 59), verapamil (*n* = 32), oligomycin (*n* = 27) and no-stimulation (*n* = 22).

To assess the effect of carbachol (CCh), an IP_3_ generating agonist, stimulation on NFATc3 translocation, we transfected INS-1 cells with GFP-tagged NFATc3 construct and loaded the cells with Fura-2 for simultaneous measurements of GFP-NFATc3 translocation and cytosolic Ca^2+^. Interestingly, nuclear translocation of GFP-NFATc3 was bi-phasic when the cells were stimulated with CCh for 3 min and monitored for 25 min (Figure 1A). Challenging the cells with CCh for 3 min resulted in an instant and transient cytosolic Ca^2+^ rise, which initiated the first phase of GFP-NFATc3 migration to the nucleus (Figure 1A and Video S1 (upper left)). In the first phase, the maximum rate of CCh-induced GFP-NFATc3 nuclear translocation was obtained within 10 min and then followed by a slow nuclear extrusion. After approximately 10 min, repetitive cytosolic Ca^2+^ spiking started and persisted until the end of the measurement. These Ca^2+^ spiking led to a second phase of GFP-NFATc3 translocation to the nucleus (Figure 1A,E–G). To test whether Ca^2+^ spiking in the second phase is dependent on the Ca^2+^ entry through the L-type Ca^2+^ channel, we used a well-known L-type Ca^2+^ channel inhibitor, verapamil [16]. Treatment of INS-1 cells with 10 µM verapamil did not affect CCh-induced cytosolic Ca^2+^ rise and translocation of GFP-NFATc3 to the nucleus in the first phase (Figure 1B,E,F). However, verapamil strongly reduced the amount of cytosolic Ca^2+^ spiking and caused a steady nuclear export of GFP-NFATc3 during the second phase (Figure 1B,E–G and Video S1 (upper right)). Accordingly, cytosolic Ca^2+^ spikes and nuclear GFP-NFATc3 translocation in the second phase were mediated by the L-type Ca^2+^ channel (Figure 1E,F). Next, we explored the importance of mitochondrial ATP production for the generation of repetitive cytosolic Ca^2+^ spiking in the second phase. Therefore, we treated the cells with the ATP synthase inhibitor, oligomycin. Similar as for verapamil, the transient Ca^2+^ rise in the cytosol upon CCh stimulation and the translocation of GFP-NFATc3 to the nucleus during the first phase remained unaffected by 2 µM oligomycin (Figure 1C,E,F). In contrast, the repetitive Ca^2+^ spiking and GFP-NFATc3 nuclear accumulation were strongly reduced by oligomycin during the second phase (Figure 1E–G and Video S1 (lower left)). These results indicate that a transient Ca^2+^ rise upon CCh stimulation leads to dynamic translocation of GFP-NFATc3 from the cytosol to the nucleus that is independent of mitochondrial ATP production. This first phase is followed by repetitive cytosolic Ca^2+^ spikes fueled by Ca^2+^ influx through the L-type Ca^2+^ channel that dependent on mitochondrial ATP production. 

To understand whether or not the Ca^2+^ spiking of the second phase is a result of the pre-stimulation of the cells with CCh in the first phase, we perfused the cells in the presence of 2 mM extracellular Ca^2+^ without CCh stimulation. Even without an earlier CCh stimulation, cytosolic Ca^2+^ spikes started after approximately 10 min and the number of spikes gradually increased over time, causing rapid nuclear accumulation of GFP-NFATc3 (Figure 1D–G and Video S1 (lower right)).

### 2.2. Near-UV Light Generates Cytosolic Ca^2+^ Spiking Dependent on the L-Type Ca^2+^ Channel Yielding Nuclear Import of GFP-NFATc3

**Figure 2:**
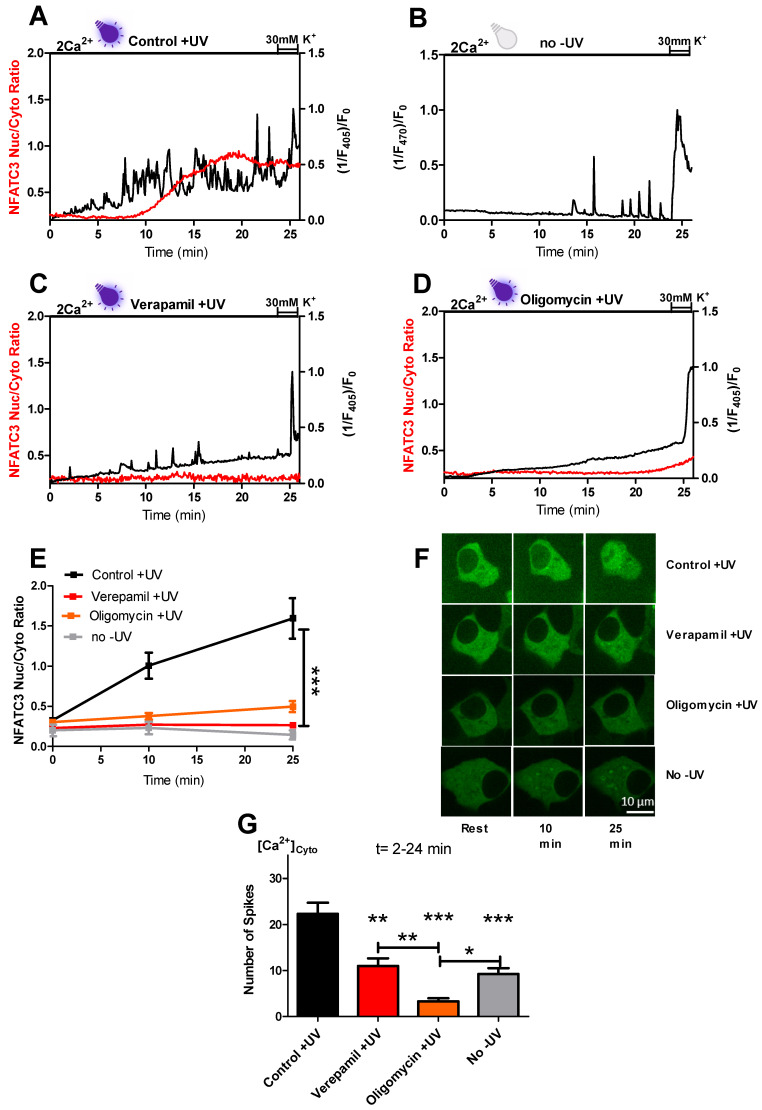
Near-UV light induces repetitive cytosolic Ca^2+^ spiking and GFP-NFATc3 translation to the nucleus via activation of L-type Ca^2+^ channels. Representative trace of repetitive cytosolic Ca^2+^ spikes (black trace) measured with Fura-2 by exciting the cells with 405 nm laser line. Simultaneous GFP-NFATc3 translocation (red trace) was assessed by exciting the cells with 488 nm laser line in the cells perfused with 2 mM extracellular Ca^2+^ solution in control +UV (**A**), no −UV (**B**), 10 µM verapamil +UV (**C**) and 2 µM oligomycin +UV (**D**). Time course of GFP-NFATc3 translocation as MEAN + SEM in control +UV (black), verapamil +UV (red), oligomycin +UV (orange), and no −UV (gray) assessed by the ratio of nucleus to the cytosol (**E**). Representative images indicate the translocation of GFP-NFATc3 in INS-1 cells. The inserted white bar represents 10 µm (**F**). Bar graphs represent the occurrence of cytosolic Ca^2+^ spiking calculated in the time point between 2 and 24 min (**G**). Significant differences were assessed using one-way ANOVA with Tukey’s multiple comparison test and presented as specific *p*-values (* *p* < 0.05, ** *p* < 0.01, *** *p* < 0.001). Control +UV (*n* = 19), verapamil +UV (*n* = 18), oligomycin +UV (*n* = 28) and no −UV (*n* = 13) for GFP-NFATc3 translocation experiment. No −UV (*n* = 28) for cytosolic Ca^2+^ measurement.

To understand what caused the cytosolic Ca^2+^ spikes and NFATc3 translocation, we focused on the protocol without CCh stimulation (Figure 2A). The cytosolic Ca^2+^ spiking trace plotted over a 65-second time range indicates the spiking pattern of Ca^2+^ (Figure S1a–c). To assess the maximum cytosolic Ca^2+^ peak via the L-type Ca^2+^ channel, which is used for normalization, 30 mM potassium treatment was performed at the end of the measurement (Figure 2A–D). Similar to Figure 1D, perfusing the cells in the presence of 2 mM external Ca^2+^ solution results in cytosolic Ca^2+^ spiking (Figure 2A,G) and the translocation of GFP-NFATc3 to the nucleus (Figure 2A,F,G and Video S2 (left)). Under resting conditions, GFP-NFATc3 shows little to no nuclear translocation, and the appearance of intensive and highly reproducible cytosolic Ca^2+^ spiking and nuclear translocation of GFP-NFATc3 indicates that the Ca^2+^ spiking and translocation are induced during the measurement. Notably, we use 405 nm laser light for excitation of Fura-2AM. This excitation wavelength is near to the ultraviolet (UV) light range and was already previously proposed to exhibit effects on cells [14]. To test the potential impact of near-UV light on the induction of Ca^2+^ spiking, we performed the same protocol using another cytosolic Ca^2+^ dye, Fluo-4 (λ_Excitation_ = 470 nm). Interestingly, exciting the cells at 470 nm light largely prevented the occurrence of a number of cytosolic Ca^2+^ spikes in comparison to cells exposed to UV light during the measurement (Figure 2B,G). To distinguish between the possible effect of Fura-2-AM loading and near-UV illumination on GFP-NFATc3 translocation, we made separate experiments with the same protocol where we loaded the cells with Fura-2 but did not illuminate them with near-UV light. Perfusing the cells for 26 min in the presence of 2 mM extracellular Ca^2+^ without near-UV light did not trigger GFP-NFATc3 translocation to the nucleus (Figure 2E,F). These results indicate that cytosolic Ca^2+^ spikes were generated due to near-UV light, leading to Ca^2+^ induced GFP-NFATc3 translocation to the nucleus. 

Next, we investigated the effect of verapamil on near-UV light induced cytosolic Ca^2+^ spiking and nuclear import of GFP-NFATc3. To address this question, in the presence of near-UV light, cells were treated with 10 µM of verapamil during the measurement (Figure 2C). Verapamil treatment strongly reduced the count of near-UV light induced cytosolic Ca^2+^ spiking (Figure 2G) and inhibited the GFP-NFATc3 translocation to the nucleus (Figure 2E,F and Video S2 (middle)) identifying L-type Ca^2+^ channels to be the target of near-UV light triggering cytosolic Ca^2+^ spiking. However, verapamil treatment did not further inhibit the amount of Ca^2+^ spiking without near-UV light induction (Figure S2f).

To clarify whether or not ATP production is required for near-UV light induced cytosolic Ca^2+^ spiking and GFP-NFATc3 translocation, we perfused INS-1 cells with 2 µM of oligomycin in 2 mM extracellular Ca^2+^ buffer (Figure 2D). Oligomycin treatment eliminated cytosolic Ca^2+^ spiking (Figure 2G) and GFP-NFATc3 migration to the nucleus (Figure 2E,F and Video S2 (right)). These data indicate that mitochondrial ATP production is essential for near-UV light induced cytosolic Ca^2+^ spikes via the L-type Ca^2+^ channel.

### 2.3. Near-UV Light Induced ROS Production Is Responsible for Ca^2+^ Spiking and NFATc3 Translocation

**Figure 3:**
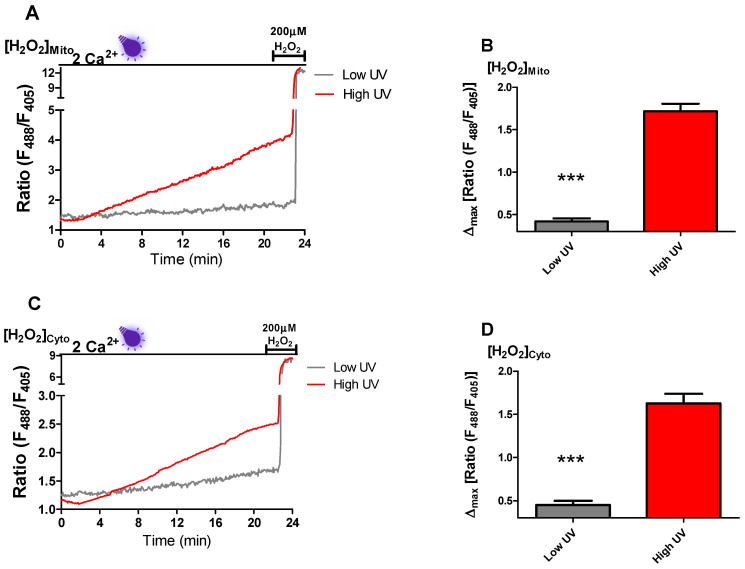
High near-UV light induces mitochondrial and cytosolic ROS production. Representative traces show the production of H_2_O_2_ in mitochondria (**A**) and cytosol (**C**) following the excitation of cells with 5 mW (low-UV) and 20 mW of a 405 nm laser line (high-UV). Right panels represent MEAN + SEM of H_2_O_2_ levels in mitochondria (**B**) and cytosol (**D**) after 20 min exposure to low-UV (gray) and high-UV (red). Significant differences were assessed via unpaired *t*-test and presented as specific *p*-values (*** *p* < 0.001). Low UV (*n* = 24) high UV (*n* = 43) in mitochondria and low UV (*n* = 20) high UV (*n* = 30) in cytosol.

It has been reported previously that UV light induces ROS production [14]. Moreover, ROS production was associated with cytosolic Ca^2+^ spiking [13]. Consequently, we investigated whether near-UV light induced Ca^2+^ spiking in INS-1 cells is the result of ROS production. For this purpose, we examined mitochondrial and cytosolic ROS levels using recently developed ultra-sensitive genetically encoded sensors mitoHyper7 and cytoHyper7 [17]. To determine the ROS production induced by near-UV light, the same experimental setup was utilized (see methods) as we used in previous experiments, and an additional 2 min 200 µM H_2_O_2_ treatment was performed to assess the saturation level of the sensor. In our experiment, 20 mW near-UV light irradiation (high-UV) led to a steady increase in both mitochondrial and cytosolic ROS production (Figure 3A,C). However, 5 mW near-UV light (low-UV) had only a very minor effect on mitochondrial and cellular ROS production (Figure 3A–D). To assess statistical comparison, we calculated near-UV light-induced maximum ROS production between high-UV and initial basal low-UV induction. High UV light induction led to a significant increase in ROS production in the mitochondria and cytosol (Figure 3B,D).

**Figure 4:**
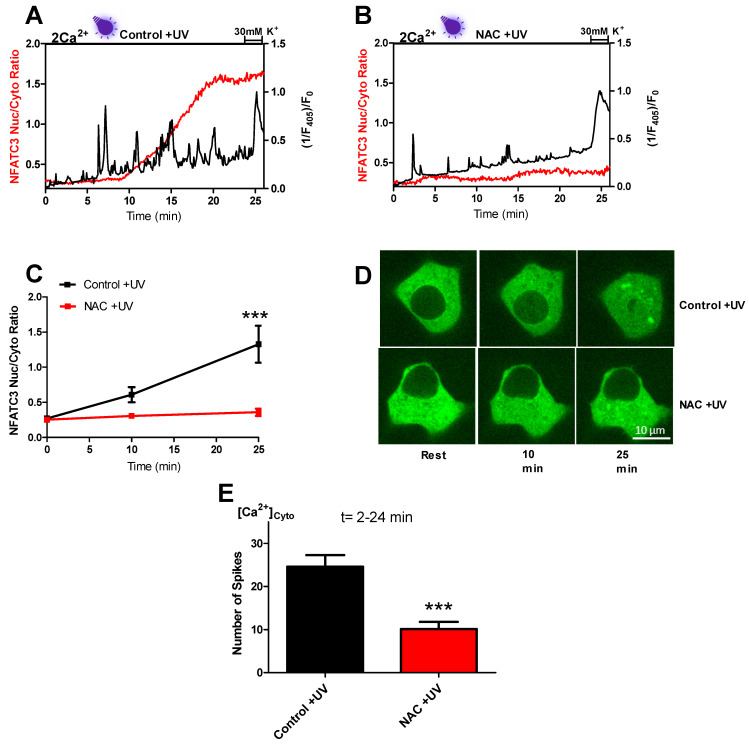
Near-UV light induced cytosolic Ca^2+^ spiking, and GFP-NFATc3 translocation to the nucleus is diminished by the application of a ROS scavenger. Representative trace of repetitive cytosolic Ca^2+^ spiking (black trace) measured with Fura-2 by exciting the cells with 405 nm laser line. Simultaneous GFP-NFATc3 translocation (red trace) was assessed by exciting the cells with 488 nm laser line in the cells perfused with 2 mM extracellular Ca^2+^ solution in control +UV (**A**) and NAC +UV (1 mM) (**B**). Time course of GFP-NFATc3 translocation as MEAN ± SEM in control +UV (black) and NAC +UV (red) assessed by the ratio of nucleus to the cytosol (**C**). Representative images indicate the translocation of GFP-NFATc3 in INS-1 cells. The inserted white bar represents 10 µm (**D**). Bar graphs represent the occurrence of cytosolic Ca^2+^ spikes calculated in the time point between 2 and 24 min (**E**). Significant differences were assessed via unpaired *t*-test and presented as specific *p*-values (*** *p* < 0.001). Control +UV (*n* = 18) and NAC (*n* = 33).

Next, we tested whether near-UV light induced cytosolic Ca^2+^ spiking and whether GFP-NFATc3 translocation is eliminated by ROS scavengers. We treated INS-cells with the well-known ROS scavenger N-acetylcysteine (NAC) [18]. Pre-incubation of the cells for 30 min with 1 mM NAC significantly reduced the amount of near-UV light-triggered cytosolic Ca^2+^ spiking (Figure 4A,B,E) and prevented nuclear translocation of GFP-NFATc3 (Figure 4A–D and Video S3). 

### 2.4. Subplasmalemmal Mitochondria Control the Near-UV Light-Induced Ca^2+^ Spiking and GFP-NFATc3 Translocation to the Nucleus by Ca^2+^ Buffering

**Figure 5:**
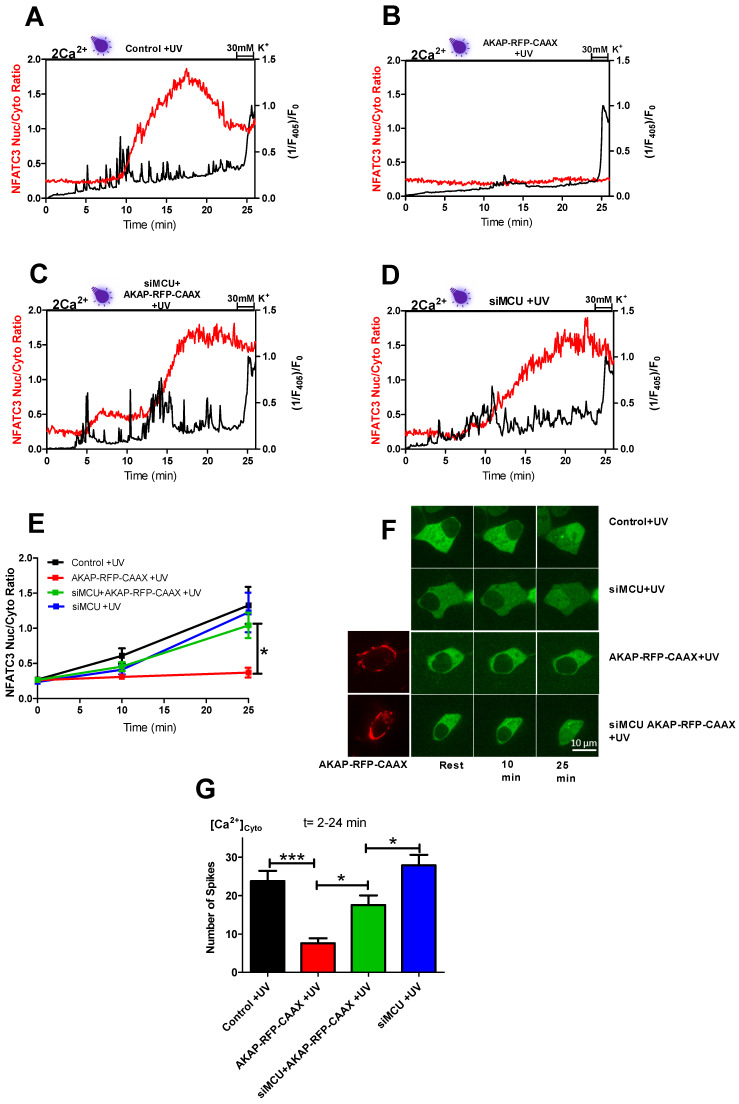
Effect of an enhancement of subplasmalemmal mitochondria’s Ca^2+^ buffering ability on near-UV light-induced cytosolic Ca^2+^ spiking and GFP-NFATc3 translocation to the nucleus. Representative trace of repetitive cytosolic Ca^2+^ spikes (black trace) measured with Fura-2 by exciting the cells with a 405 nm laser line. Simultaneous GFP-NFATc3 translocation (red trace) was assessed by exciting the cells with a 488 nm laser line in the cells perfused with 2 mM extracellular Ca^2+^ solution in control +UV (**A**), AKAP-RFP-CAAX +UV (**B**), siMCU+AKAP-RFP-CAAX +UV (**C**) and siMCU +UV (**D**). Time course of GFP-NFATc3 translocation as MEAN ± SEM in control +UV (black), AKAP-RFP-CAAX +UV (red), siMCU+AKAP-RFP-CAAX (green), and siMCU (blue) assessed by the ratio of nucleus to the cytosol (**E**). Representative images indicate the translocation of GFP-NFATc3 in INS-1 cells. The inserted bar represents 10 µm (**F**). Bar graphs represent the occurrence of cytosolic Ca^2+^ spiking calculated in the time point between 2 and 24 min (**G**). Significant differences were assessed using one-way ANOVA with Tukey’s multiple comparison test and presented as specific *p*-values (* *p* < 0.05, *** *p* < 0.001) control +UV (*n* = 18), AKAP-RFP-CAAX +UV (*n* = 30), siMCU+AKAP-RFP-CAAX +UV (*n* = 33) and siMCU +UV (*n* = 20).

Next, we elucidated whether the ROS production or cytosolic Ca^2+^ spiking drives the nuclear translocation of NFATc3 in response to near-UV light exposure. To approach this question, we came up with a strategy to buffer the Ca^2+^ spiking through the L-type Ca^2+^channel by increasing the proximity between mitochondria and the sub-plasma membrane region. This was achieved by expressing the AKAP-RFP-CAAX construct [19,20,21] in INS-1 cells, which positioned mitochondria in the sub-PM region (Figure 5F, red images). By expressing AKAP-RFP-CAAX together with GFP-NFATc3 in Fura-2-loaded INS-1 cells, we monitored GFP-NFATc3 translocation and cytosolic Ca^2+^ spiking simultaneously. In AKAP-RFP-CAAX-expressing cells, the number of near-UV light-triggered cytosolic Ca^2+^ spikes were strongly reduced (Figure 5G). Close contact of mitochondria to the plasma membrane might lead to increased mitochondrial Ca^2+^ buffering capacities for entering Ca^2+^ via the L-type Ca^2+^ channel that normally fuels the near-UV light-induced Ca^2+^ spiking. Moreover, Ca^2+^ driven translocation of the GFP-NFATc3 was diminished in these cells (Figure 5B,E–G Video S4 (upper right)). Next, we intended to challenge our hypothesis that subplasmalemmal mitochondrial Ca^2+^ buffering of entering Ca^2+^ accounts for the prevention of near-UV light-triggered Ca^2+^ spiking and GFP-NFATc3 nuclear translocation. Therefore, we hampered the Ca^2+^ buffering ability of mitochondria by transient siRNA-mediated knockdown of the mitochondrial calcium uniporter (MCU) in AKAP-RFP-CAAX-expressing cells. The count of spikes was significantly increased by MCU knockdown in AKAP-RFP-CAAX-expressing cells comparing to AKAP-RFP-CAAX (Figure 5G). Hence, GFP-NFATc3 translocation was also rescued in siMCU+AKAP-RFP-CAAX-expressing cells (Figure 5E–G and Video S4 (lower left)). In addition, we checked whether MCU knockdown itself has any effect on cytosolic Ca^2+^ spiking and GFP-NFATc3 translocation. Cells treated with siRNA against MCU showed similar cytosolic Ca^2+^ spiking and translocation as control cells (Figure 5D–G and Video S4 (lower right)). Moreover, a frequency of one spiking per minute was needed to start the translocation of NFATc3 under these conditions (Figure S3a–d). However, we noticed a co-dependence on the area under the curve (AUC) of Ca^2+^ spiking; if the AUC was smaller, more frequency was required to start the translocation. Additionally, we confirmed that in the absence of near-UV light, no repetitive cytosolic Ca^2+^ spiking or GFP-NFATC3 translocation to the nucleus occured in these conditions (Figure S2a–f).

To test whether mitochondrial Ca^2+^ buffering ability affects near-UV light-triggered mitochondrial or cytosolic ROS production, we determined near-UV light induced ROS production in cells with transient MCU knockdown, cells expressing AKAP-RFP-CAAX and MCU knockdown in AKAP-RFP-CAAX-expressing cells. Maximum ROS production under high near-UV irradiation did not significantly change between the control, MCU siRNA-treated, AKAP-RFP-CAAX-expressing and MCU knockdown in AKAP-RFP-CAAX-expressing cells (Figure S4a–d). These results indicate that the reduction in the near-UV light-induced cytosolic Ca^2+^ spiking and nuclear import of GFP-NFATc3 in AKAP-RFP-CAAX cells is related to mitochondria’s subplasmalemmal Ca^2+^ buffering but not alterations in cytosolic or mitochondrial ROS production.

## 3. Discussion

Among other NFAT members, NFATc3 has the highest expression level in human and mouse pancreatic islets [5] and plays an important role in pancreatic β-cells [10]. To gain more insight into the nuclear translocation mechanism of NFATc3, we investigated the translocation kinetics of NFATc3 in two distinct phases. In the first phase, CCh stimulation leads to intracellular Ca^2+^ store mobilization, resulting in cytoplasmic Ca^2+^ rise (Figure 1A), which, in turn, activates NFATc3 migration to the nucleus. However, phase two was characterized by strong cytosolic Ca^2+^ spikes leading to further migration of NFATc3 to the nucleus (Figure 1A,E,F). Interestingly, the Ca^2+^ spiking and NFATc3 translocation in the second phase were eliminated by verapamil treatment (Figure 1B,G). In line with the previous study [22], these results indicate that NFATc3 translocation in the first phase was the result of endoplasmic reticulum (ER) Ca^2+^ release, which likely triggers Ca^2+^ influx via calcium release-activated channels (CRAC) [4]. In compliance with this assumption, verapamil treatment did not affect cytosolic Ca^2+^ rise and NFATc3 translocation in the first phase but in the second phase. 

Our findings that both the Ca^2+^ spiking and NFATc3 translocation in the second phase were sensitive to mitochondrial ATP synthase inhibitor oligomycin are in agreement with the general expectation that the CCh-induced Ca^2+^ release stimulates mitochondrial ATP production that, in turn, blocks the K_ATP_ channel and depolarizes the plasma membrane, thus promoting the opening of the L-type Ca^2+^ channel and the subsequent nuclear translocation of GFP-NFATc3 (Figure 1C,E–G). However, to our surprise, cells without CCh stimulation showed comparable repetitive Ca^2+^ spiking and robust translocation of NFATc3 to the nucleus (Figure 1D–G), thus eliminating the involvement of CCh stimulation on the Ca^2+^ spiking and NFATc3 translocation during the second phase.

Therefore, we focused on an alternative trigger for cytosolic Ca^2+^ spiking which drives NFATc3 translocation to the nucleus. In our spinning disc confocal microscopic setup, parallel measurements of cytosolic Ca^2+^ using Fura-2 and GFP tagged NFATc3 translocation require the use of near-UV excitation with the high-energy 405 nm wavelength. We assumed that Ca^2+^ spiking could be the result of near-UV light. Indeed, in the presence of near-UV light, we observed repetitive cytosolic Ca^2+^ spikes which led to robust migration of NFATc3 to the nucleus (Figure 2A,E–G). Previous studies support our results of near-UV light-induced cytosolic Ca^2+^ oscillations and NFAT migration to the nucleus in different cell lines [12,14]. Since INS-1 cells show Ca^2+^ spiking under basal conditions (Figure 2B,G and Figure S3a,f), we compared the amount of Ca^2+^ spiking of cells exposed to near-UV light to non-UV stimulated control cells. Interestingly, Ca^2+^ spiking in non-UV cells was halved compared to near-UV light-exposed cells (Figure 2G). NFATc3 translocation to the nucleus did not occur in cells without near-UV light exposure (Figure 2B,E–G), indicating that near-UV light is required to boost the Ca^2+^ spikes for migration of NFAtc3 to the nucleus.

Furthermore, inhibition of L-type Ca^2+^ channels with verapamil decreased the amount of UV light-induced Ca^2+^ spiking to the level of non-UV used cells, and these Ca^2+^ spiking were not enough to drive NFATc3 translocation to the nucleus (Figure 2C,E–G). Thus, our findings confirm the published data on the impact of the frequency of Ca^2+^ spiking on NFAT translocation to the nucleus [9]. Additionally, these results highlight the source of near-UV light induced Ca^2+^ spikes as well as the importance of a tight threshold in the amount of Ca^2+^ spiking to fire NFATc3 migration to the nucleus. Next, we investigated whether basal ATP production is required to initiate near-UV light-induced cytosolic Ca^2+^ spikes for NFATc3 translocation. Interestingly, oligomycin treatment eliminates the spiking as well as nuclear translocation of NFATc3, indicating that mitochondrial basal ATP production is indispensable for UV light-induced Ca^2+^ spiking (Figure 2D–G). However, the exact mechanism of ATP dependency of near-UV light-induced Ca^2+^ spiking and NFATc3 translocation is not completely clear and needs further attention. 

To shed light on the mechanism of near-UV light-generated Ca^2+^ spiking, we checked mitochondrial and cytosolic ROS production in INS-1 cells. In line with a previous study [14], both in mitochondria and cytosol, we observed a steady increase in ROS production in the presence of near-UV light (Figure 3A–D). Surprisingly, near-UV light induced repetitive Ca^2+^ spiking started mostly after 7 min of exposure, indicating that a certain threshold of ROS levels has to be reached to trigger L-type Ca^2+^ channel activity and subsequent NFATc3 translocation. Interestingly, a former study indicated that moderate ROS levels induce β-cell proliferation [23]. NFATc3 translocation to the nucleus increases the expression of genes responsible for cell proliferation [5,10]. It is tempting to speculate that β-cells have the necessity to accumulate a ROS threshold (e.g., during high metabolic activity) to activate NFATc3 driven proliferation.

Migration of NFATc3 to the nucleus happens in a sequence of events that starts with near-UV light-induced ROS production. Consequently, we assumed that ROS scavenging prevents the signaling cascade. Indeed, NAC, a well-known ROS scavenger, decreased the amount of near-UV light-induced cytosolic Ca^2+^ spiking (Figure 4A,E). In support of our findings, another study also reported reduced UV light-generated cytosolic Ca^2+^ oscillations upon NAC treatment [14]. In response to hampered cytosolic Ca^2+^ spikes, NFATc3 migration to the nucleus was also diminished by NAC treatment (Figure 4B–E). These results support our assumption that near-UV light-induced ROS production leads to cytosolic Ca^2+^ spiking through the L-type Ca^2+^ channel, and these spikes drive NFATc3 translocation to the nucleus.

Having established that ROS induced repetitive cytosolic Ca^2+^ spikes, which happen via the L-type Ca^2+^ channels and drive the NFATc3 translocation to the nucleus, we wanted to distinguish between the direct effect of ROS and ROS-induced cytosolic Ca^2+^ spiking on NFATc3 translocation. To do this, we came up with a hypothesis that if we buffer Ca^2+^ spikes, which originate from L-type Ca^2+^ channels at the plasma membrane, but keep the same ROS levels, we would not see the NFATc3 translocation and Ca^2+^ oscillations. To test this idea, we positioned mitochondria to the sub-plasma membrane region using the AKAP-RFP-CAAX construct [19,20,21] to buffer local Ca^2+^ spiking. Interestingly, AKAP-RFP-CAAX-expressing cells did not alter ROS production (Figure S4a–d) in response to near-UV light stimulation, but they eliminated near-UV light-induced Ca^2+^ spiking as well as NFATc3 translocation (Figure 5B,E–G), thus supporting our hypothesis that ROS-induced cytosolic Ca^2+^ spiking, and not ROS itself, is required for NFATc3 translocation to the nucleus. To further validate this point, we knocked down MCU protein in AKAP-RFP-CAAX-expressing cells to diminish mitochondrial Ca^2+^ uptake and buffering, which resulted in the reappearance of cytosolic Ca^2+^ spiking and rescued migration of NFATc3 to the nucleus (Figure 5C,E–G). A recently published paper revealed that 18% of the sub-PM area is occupied by mitochondria in β-cells [24]. However, MCU KD alone did not affect UV light-induced Ca^2+^ spiking and NFATc3 translocation (Figure 5D–G). This could be because of the free motility of the sub-PM located mitochondria. The effect of near-UV induction on mitochondrial motility is not known. However, long-term intense blue light does not affect the sub-PM distributed mitochondrial density. In contrast, Ca^2+^ entry via the L-type Ca^2+^ channel significantly decreases the sub-PM located mitochondria from 18% to 13% in MIN6 cells [24]. Thus, it is tempting to speculate that in control or siMCU cells, sub-PM localized mitochondria are not able to buffer UV light-induced Ca^2+^ spiking to a great extent because of the Ca^2+^ regulated mitochondrial redistribution. 

In conclusion, this study reveals that near-UV light induced ROS production leads to repetitive cytosolic Ca^2+^ spiking via the activation of the L-type Ca^2+^ channel, triggering NFATc3 translocation to the nucleus. Moreover, we presented mitochondria as Ca^2+^ sinks by tagging them to the cell membrane, where mitochondria buffered cytosolic Ca^2+^ rises due to L-type Ca^2+^ channel activity and thereby prevented NFATc3 translocation. In addition, this study highlights the sensitivity of the pancreatic β-cells to near-UV light ranges frequently used in live-cell imaging for excitation of blue fluorescent proteins as well as cell-permeable dyes such as Fura-2.

## 4. Materials and Methods

### 4.1. Cell Culture and Transfection

The INS-1 832/13 (INS-1) cells were a generous gift from Prof. Dr. Claes B. Wollheim and Dr. Françoise Assimacopoulos-Jeannet (University Medical Center, Geneva, Switzerland). The INS-1 cells were cultured in RPMI 1640 containing 11 mM glucose (PubChem CID: 5793) supplemented with 10 mM HEPES (PubChem CID: 23831), 10% fetal calf serum (FCS), 1 mM sodium pyruvate (PubChem CID: 23662274), 50 μM β-mercaptoethanol (PubChem CID: 1567), 1% (*v*/*v*) Pen Strep^®^ (ThermoFischer, Vienna, Austria; 10.000 U/L) 1.25 μg/mL Amphotericin B (ThermoFischer, Vienna, Austria; 250 μg/mL). Cells were used between passage numbers 53 and 68.

For all microscopic experiments, cells were plated on 30 mm glass coverslips in 6-well plates and transfected at 50–60% confluency with AKAP-RFP-CAAX (1 μg/well), GFP-NFATc3 (2 μg/well), mitoHyper7, and cytoHyper7 (1 μg/well) DNA constructs alone or with MCU siRNA (siRNA sequence: 5′-AAA GUC UCG UUU CGA CCU ATT-3′) by using 3 μL TransFast transfection reagent (Promega, Madison, WI, USA) in 1 mL of serum and antibiotic-free medium for 12–14 h. After that, transfection media was replaced with 2 mL of full culture medium. All experiments were performed 40-45 h after transfection.

### 4.2. Quantitative PCR

Total mRNA was isolated using the RNeasy® Mini Kit (Qiagen, Hilden, Germany), and reverse transcription was performed using Applied Biosystems High-Capacity cDNA Reverse Transcription kit (Thermo Fisher Scientific Baltics UAB, Vilnus, Lithuania). qPCR was performed using Promega GOTaq^®^ qPCR Master Mix (Madison, WI, USA). Knock-down efficiency of rMCU (Figure S5) was determined using specific primers for rMCU (Forward: GCGTTGCCATCTATTCCCCA; reverse: TGGCTCAGGAGGTCTCTCTTT) and normalized to rGAPDH (Forward: TCTACATGTTCCAGTATGACTC; reverse: GCATCACCCCATTT GATG).

### 4.3. Buffers

Prior to experiments, cells were adjusted to room temperature with experimental buffer (EB): 2 mM Ca^2+^, 138 mM NaCl, 1 mM MgCl_2_, 5 mM KCl, 10 mM HEPES, 2.6 mM NaHCO_3_, 0.44 mM KH_2_PO_4_, amino acid and vitamin mix, 10 mM glucose, 2 mM L-glutamine, 1% penicillin/streptomycin, 1.25 μg/mL amphotericin B, pH adjusted to 7.4. All experiments were performed in following buffers 2CaNa buffer (2 mM CaCl_2_, 138 mM NaCl, 1 mM MgCl_2_, 5 mM KCl, 10 mM Hepes, 10 mM D-glucose, pH 7.4) and 2Ca30K buffer (2 mM CaCl_2_, 113 mM NaCl, 1 mM MgCl_2_, 30 mM KCl, 10 mM Hepes, 10 mM D-glucose, pH 7.4).

### 4.4. Live Cell Imaging Experiments

If not stated otherwise, all experiments were performed with a Zeiss array confocal laser scanning microscope (Axio Observer.Z1 from Zeiss, Gottingen, Germany) by using 100x objective lens (Plan-Fluor x100/1.45 Oil, Zeiss, Germany). This was equipped with a motorized filter wheel (CSUX1FW, Yokogawa Electric Corporation, Tokyo, Japan) on the emission side, an AOTF-based laser merge module for the 405, 445, 473, 488, 514, and 561 nm laser lines (Visitron Systems), and a Nipkow-based confocal scanning unit (CSU-X1, Yokogawa Electric Corporation). Data acquisition and control of the fluorescence microscope were performed using Visiview 4.2.01 (Visitron, Puchheim, Germany). 

### 4.5. GFP-NFATc3 Translocation and Ca^2+^ Experiments with UV Light

GFP-NFATc3 translocation experiments were performed with the GFP-NFATc3 construct (gift from Mark L. Dell’Acqua, Department of Pharmacology, University of Colorado School of Medicine, Aurora). If not stated otherwise, all GFP-NFATc3 translocation experiments were performed using the following imaging parameters. GFP-NFATc3 was excited with 488 (30 mW) nm laser lines every 5 seconds for a 500-millisecond exposure time, and emissions were acquired at 510 nm. To stimulate the cells with UV light and follow the simultaneous changes in [Ca^2+^]_cyto_, cells were loaded with 3.3 µM Fura-2 in EB for 30 min and excited with 405 nm laser lines (20 mW) every 5 s for a 500-millisecond exposure time and emissions were acquired at 510 nm by using a charged CCD camera (CoolSNAP-HQ, Photometrics, Tucson, AZ, USA). VisiView acquisition software (Universal Imaging, Visitron Systems) was used to acquire the [Ca^2+^]_cyto_ and translocation of GFP-NFATc3 from the cytosol to the nucleus. Background subtracted GFP-NFATc3 fluorescence ratio of the nucleus to cytosol region was used as a readout, where min 0 shows the resting ratio of GFP-NFATc3, min 10 shows the maximum translocation ratio of GFP-NFATc3 in the 0 to 10 min time interval whereas min 25 indicates the maximum translocation ratio of GFP-NFATc3 in the 20 to 25 min time interval.

To check bi-phasic GFP-NFATc3 translocation, Fura-2-AM loaded cells were stimulated with 100µM CCh in the presence of 2 mM extracellular Ca^2+^ and changes in [Ca^2+^]_cyto_ and translocation of GFP-NFATc3 were recorded. The amount of cytosolic Ca^2+^ spiking was calculated between min 10 and 25.

Inhibition of [Ca^2+^]_cyto_ spiking and GFP-NFATc3 translocation was performed by perfusing the cells in the presence of 2 µM oligomycin (Sigma-Aldrich, Vienna, Austria) and 10 µM verapamil (Sigma-Aldrich, Vienna, Austria) during the experiment or 30 min preincubation with 1 mM NAC (Sigma-Aldrich, Vienna, Austria). The number of cytosolic Ca^2+^ spikes was calculated between min 2 and 24.

To assess GFP-NFATc3 translocation without UV, cells were loaded with 3.3 µM Fura-2-AM in EB for 30 min. In the presence of 2 mM extracellular Ca^2+^, a 488 nm excitation laser was used to track GFP-NFATc3 migration alone for 26 min.

### 4.6. Analysis of Cytosolic Ca^2+^ Spiking

First, cytosolic Ca^2+^ traces were background corrected using background ROI intensities. Furthermore, the standard deviation (SD) of basal Ca^2+^ signals was calculated to estimate the signal-to-noise ratio. First, peaks were identified by a change from positive to the negative slope between measurement points. As a second characteristic, intensity changes between time points of positive to negative slope changes and negative to positive slope changes had to exhibit 5-fold higher values than the basal SD values to be counted as a valid signal and identified as a Ca^2+^ spike. 

### 4.7. Mitochondrial and Cytosolic ROS Measurements

Mitochondrial and cytosolic ROS measurements were performed with the recently developed genetically encoded H_2_O_2_ sensors mitoHyper7 and cytoHypher targeted to the matrix or cytosol, respectively [17]. Both sensors were excited with 405 and 488 nm laser lines, and emissions were acquired at 510 nm. To record the baseline, cells were excited at 5 mW with 405 and 488 nm laser lights every 5 seconds with a 500-millisecond exposure for 2 min. At the end of min 2, excitation with the 405 and 488 nm lasers was increased to 20 milliwatts and measurement was performed for 22 more min in these settings. At the end of min 22, cells were stimulated with 200 µM of H_2_O_2_ to obtain the saturation ratio of the sensors. Control cells were excited with 5 mW 405 and 488 nm laser lights every 5 seconds with 500-millisecond exposure for 22 min where ROS production did not occur. The background subtracted maximum UV light-induced H_2_O_2_ ratio of 488/405 was subtracted from the baseline to obtain the change in ROS production at the end of 20 min with 20 mW repetitive UV induction.

### 4.8. Cytosolic Ca^2+^ Measurements without UV Light

Cytosolic Ca^2+^ measurement without UV light was performed with an Olympus IX73 inverted microscope. This microscope was equipped with an UApoN340 40× oil immersion objective (Olympus, Tokyo, Japan) and a CCD Retiga R1 camera (Q-imaging, Vancouver, BC, Canada). LedHUB^®^ (Omnicron, Rodgau, Germany) equipped with 340, 385, 455, 470, and 550 nm LEDs. A GFP (GFP-3035D, Semrock, Henrietta, NY, USA) filter set was used for illumination of Fluo-4. Visiview 4.2.01 (Visitron, Puchheim, Germany) was used for the data acquisition. Alternatively, an AnglerFish F-G/O (Next Generation Fluorescence Imaging/NGFI (www.ngfi.eu), Graz, Austria) was used for data acquisition. Subsequent data analysis was performed in ImageJ (NIH, Bethesda, MD, USA) and Excel (Microsoft, Redmond, WA, USA). Both microscopes were equipped with an automatic perfusion system PS-9D (NGFI).

To avoid usage of UV light, cells were loaded with 3.3 µM Fluo-4 in EB for 30 min. On the microscope, cells were perfused with 2 mM Ca^2+^ buffer for 24 min and 2-min 30 mM K^+^ buffer to get maximum uptake through the L-type Ca^2+^ channels. The depolarization induced maximum Ca^2+^ uptake via L-type Ca^2+^ channel is used for the normalization of Fura-2. The number of cytosolic Ca^2+^ spikes was calculated from min 2 to min 24.

### 4.9. Data Analysis

The data shown above were acquired from three different days and represent the mean ± SEM. The number of single cells is represented as *n* = cell number in each figure legend. Single cells were used for the statistical analysis, where Student’s t-test and analysis of variance (ANOVA) with Tukey post hoc test were performed. GraphPad Prism software version 5.04 (GraphPad Software, San Diego, CA, USA) and Microsoft Excel (Microsoft) were used for the analysis, calculation, and representation of the data.

## Data Availability

The data presented in this study are available on request from the corresponding author.

## Supplementary Materials

The following are available online at https://www.mdpi.com/article/10.3390/ijms22158189/s1.
