# Supplementary material for: Near-UV Light Induced ROS Production Initiates Spatial Ca2+ Spiking to Fire NFATc3 Translocation
Source: Int J Mol Sci. 2021 Jul 30;22(15):8189. doi: 10.3390/ijms22158189 (PMC8346968; doi:10.3390/ijms22158189)
Supplement: Supplementary file 1 [file ijms-22-08189-s001.zip › Moive Legends _01.pdf]

Video 1. Bi-phasic regulation of nuclear GFP-NFATc3 translocation in INS-1 cells. Representative 24-minutes-long videos show simultaneously measured cytosolic Ca^{2+} oscillations and GFPNFATc3 translocation to the nucleus in Control +UV (upper left), 10 μM Verapamil +UV (upper right), 2 μM Oligomycin +UV (lower left), and no-stimulation +UV (lower right). Cells were perfused with 2 mM extracellular Ca^{2+} solution in the presence of near UV light induction for each 5 seconds and stimulated with CCh for 3 min as indicated in the movie.

Video 2. Near UV light induces repetitive cytosolic Ca^{2+} oscillations and GFP-NFATc3 translation to the nucleus via activation of L-type Ca^{2+} channels. Representative 24-minutes-long videos show simultaneously measured cytosolic Ca^{2+} oscillations and GFPNFATc3 translocation to the nucleus in Control +UV (left), 2 μM Oligomycin +UV (right) and 10 μM Verepamil +UV (middle). Cells were perfused with 2 mM extracellular Ca^{2+} solution in the presence of near UV light induction for each 5 seconds.

Video 3. Near UV-induced cytosolic Ca^{2+} oscillations and GFP-NFATc3 translocation to the nucleus are diminished by the application of a ROS scavenger. Representative 24-minutes-long videos show simultaneously measured cytosolic Ca^{2+} oscillations and GFPNFATc3 translocation to the nucleus in Control +UV (left) and 1 mM NAC +UV (right). Cells were perfused with 2 mM extracellular Ca^{2+} solution in the presence of near UV light induction for each 5 seconds.

Video 4. Effect of an enhancement of subplasmalemmal mitochondria's Ca^{2+} buffering ability on near UV-induced cytosolic Ca^{2+} oscillations and GFP-NFATc3 translocation to the nucleus. Representative 24-minutes-long videos show simultaneously measured cytosolic Ca^{2+} oscillations and GFPNFATc3 translocation to the nucleus in Control +UV (upper left), AKAP-RFP-CAAX +UV (upper right), AKAP-RFP-CAAX + MCUsi + UV (lower left), and MCUsi +UV (lower right). Cells were perfused with 2 mM extracellular Ca^{2+} solution in the presence of near UV light induction for each 5 seconds.
