# Supplementary material for: Near-UV Light Induced ROS Production Initiates Spatial Ca2+ Spiking to Fire NFATc3 Translocation
Source: Int J Mol Sci. 2021 Jul 30;22(15):8189. doi: 10.3390/ijms22158189 (PMC8346968; doi:10.3390/ijms22158189)
Supplement: Supplementary file 1 [file ijms-22-08189-s001.zip › Suplementary File.pdf]

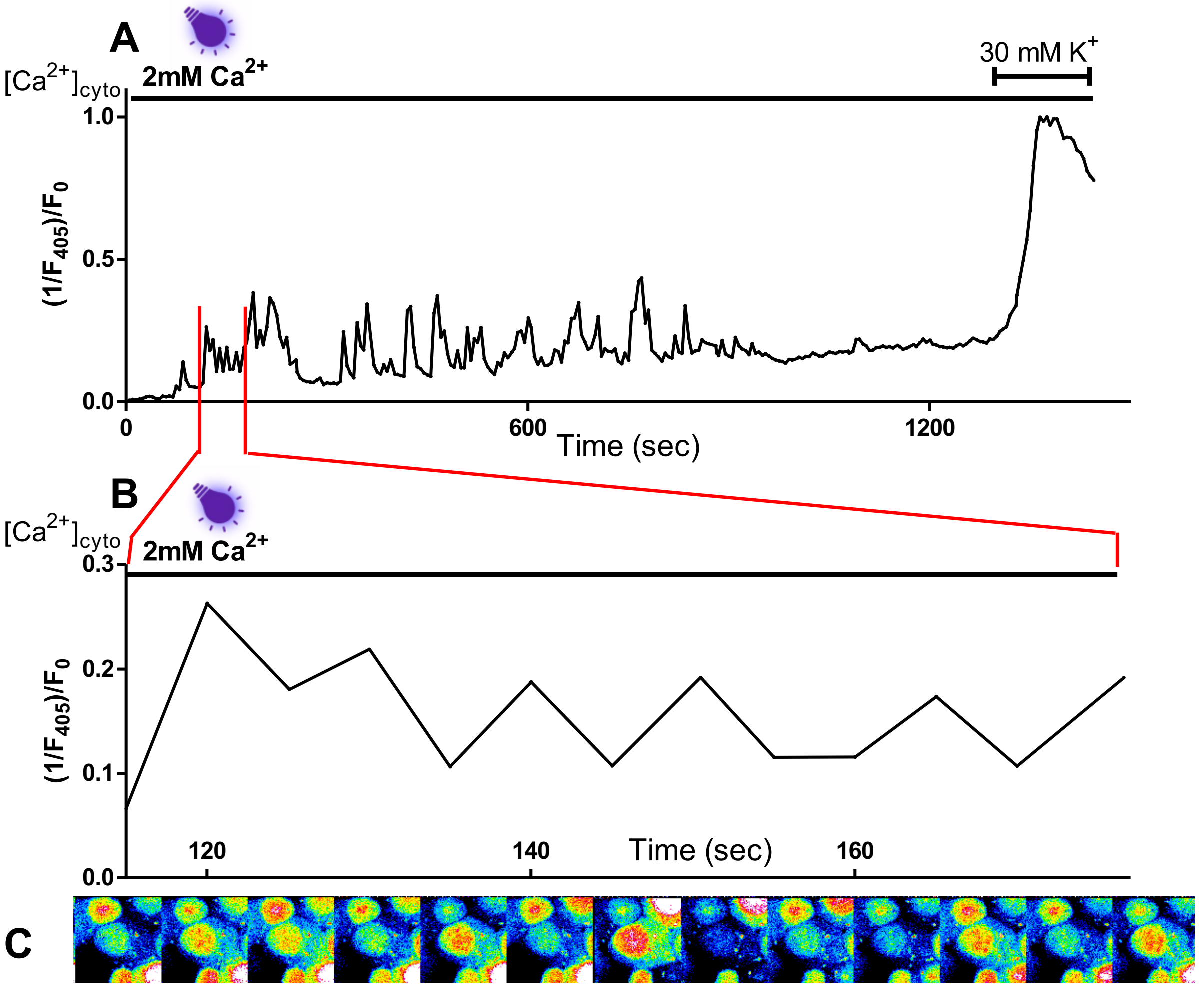

Supplementary Figure 1. Exemplary Cytosolic Ca^{+2} spiking. Representative trace of repetitive cytosolic Ca^{2+} spiking measured with Fura-2 by exciting the cells with 405 nm laser line (A). The time point between 115 to 180 seconds of trace (A) was plotted over 65 seconds (B) and the respective montage of the Ca^{2+} spiking was shown (C). The whole movie of the Ca^{2+} spiking of the same cell and respective GFPNFATc3 translocation is added to supplementary video 3 and indicated as Control +UV.

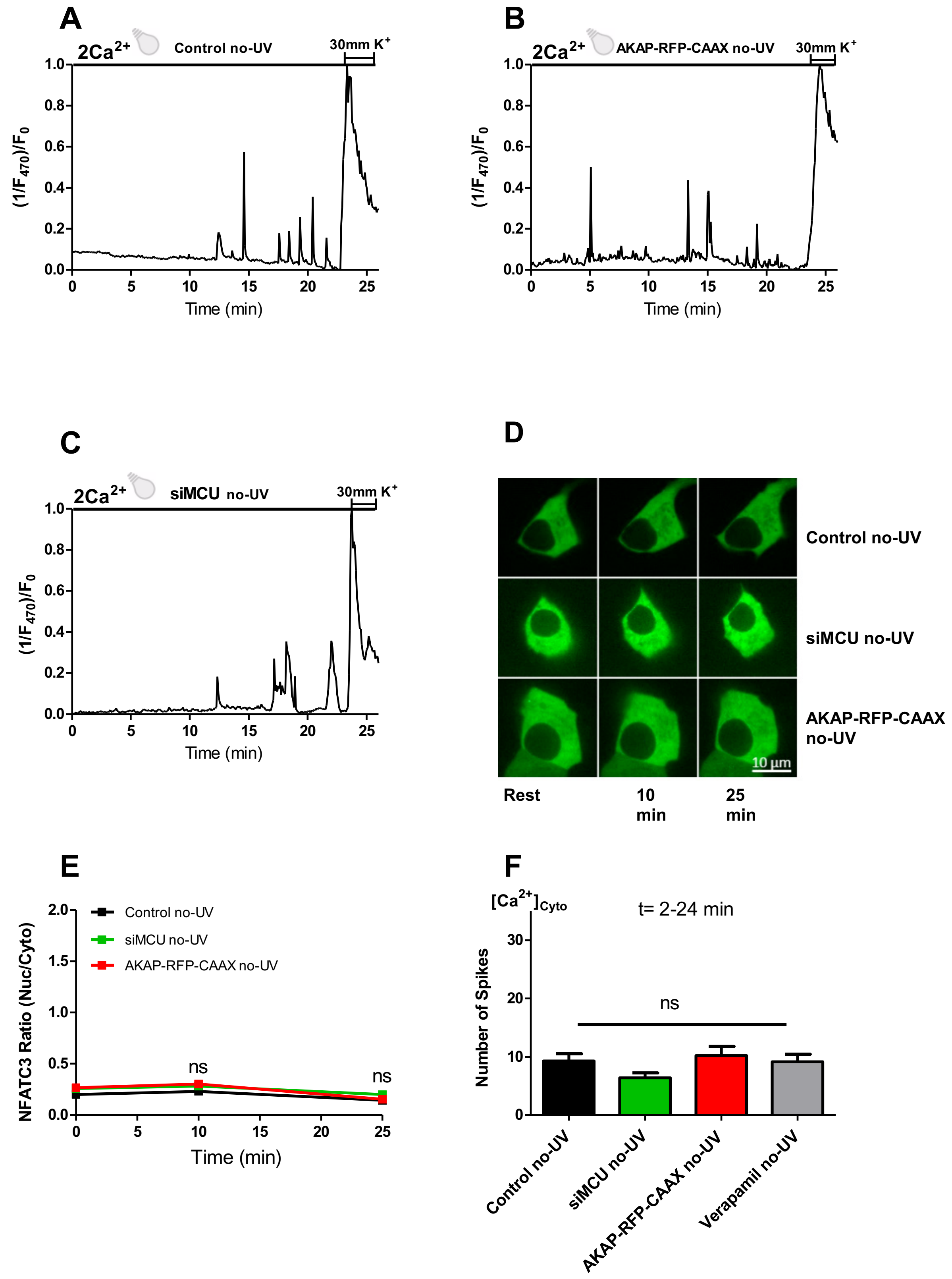

A
2Ca2+
Control no-UV
30mm K+
(1/F470)/F0
Time (min)
B
2Ca2+
AKAP-RFP-CAAX no-UV
30mm K+
(1/F470)/F0
Time (min)
C
2Ca2+
siMCU no-UV
30mm K+
(1/F470)/F0
Time (min)
D
Control no-UV
siMCU no-UV
AKAP-RFP-CAAX no-UV
10 μm
Rest
10 min
25 min
E
Control no-UV
siMCU no-UV
AKAP-RFP-CAAX no-UV
NFATC3 Ratio (Nuc/Cyto)
ns
ns
Time (min)
F
[Ca2+]Cyto
t= 2-24 min
Number of Spikes
ns
Control no-UV
siMCU no-UV
AKAP-RFP-CAAX no-UV
Verapamil no-UV

Supplementary Figure 2. Absence of UV induction keeps NFATc3 in cytosol. Representative trace of cytosolic Ca^{2+} spikes (black trace) measured with Fluo-4 by exciting the cells with 470 nm laser line in the cells perfused with 2 mM extracellular Ca^{2+} solution in Control no-UV (A), AKAP-RFP-CAAX no-UV (B) and siMCU no-UV (C). Representative images indicate the translocation of GFP-NFATc3 translocation in INS-1 cells perfused at 2mM external Ca^{2+} solution without UV (D). Time course of GFP-NFATc3 translocation as MEAN $\pm$ SEM in Control no-UV (black), AKAP-RFP-CAAX no-UV (red) and siMCU no-UV (green) assessed by the ratio of nucleus to the cytosol (E). Bar graphs represent the occurrence of cytosolic Ca^{2+} spiking calculated in the time point between the 2 and 24 minutes (F). Significant differences were assessed using one-way ANOVA with Tukey's multiple comparison test and presented as specific p-values (n.s. – not significant). Control no-UV (n=21), AKAP-RFP-CAAX no-UV (=21), siMCU no-UV (n=25) and Verapamil no-UV (n=23) for cytosolic Ca^{2+} measurement and Control no-UV (n=13), AKAP-RFP-CAAX no-UV (=11), siMCU no-UV (n=10) for GFP-NFATc3 translocation.

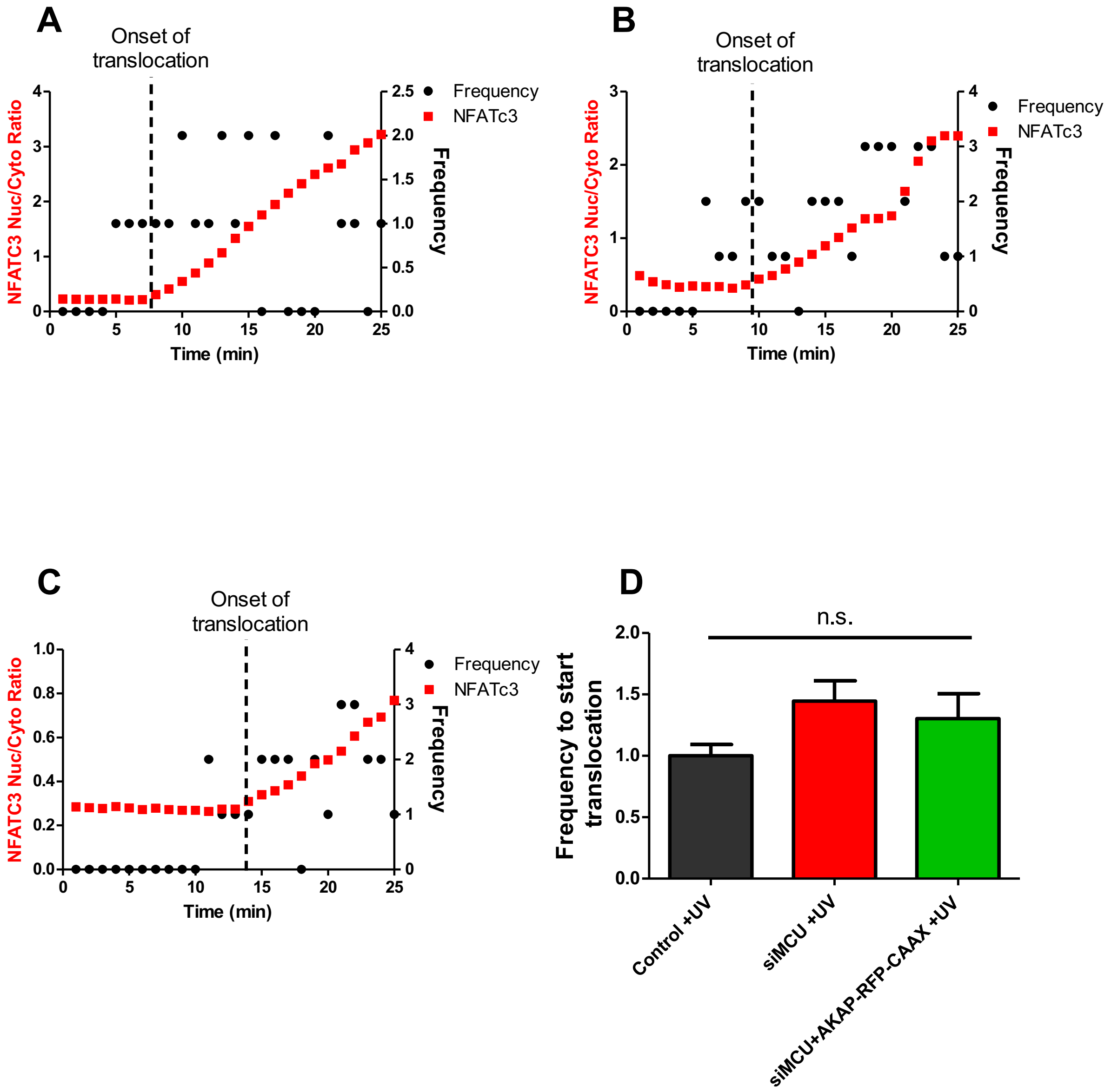

Supplementary Figure 3. Dependency of NFATc3 translocation to number of cytosolic Ca^{2+} Spiking. Cytosolic Ca^{2+} spiking frequency of 3 minutes leading to the onset of NFATc3 translocation to the nucleus was quantified in Control +UV (A), siMCU +UV (B) and siMCU+AKAP-RFP-CAAX +UV (C). Bar graphs represent the frequency of Ca^{2+} spiking (D). Significant differences were assessed using one-way ANOVA with Tukey's multiple comparison test and presented as specific p-values (n.s. – not significant). Control +UV (n=18), siMCU +UV (n=12) and AKAP-RFP-CAAX +UV (=21).

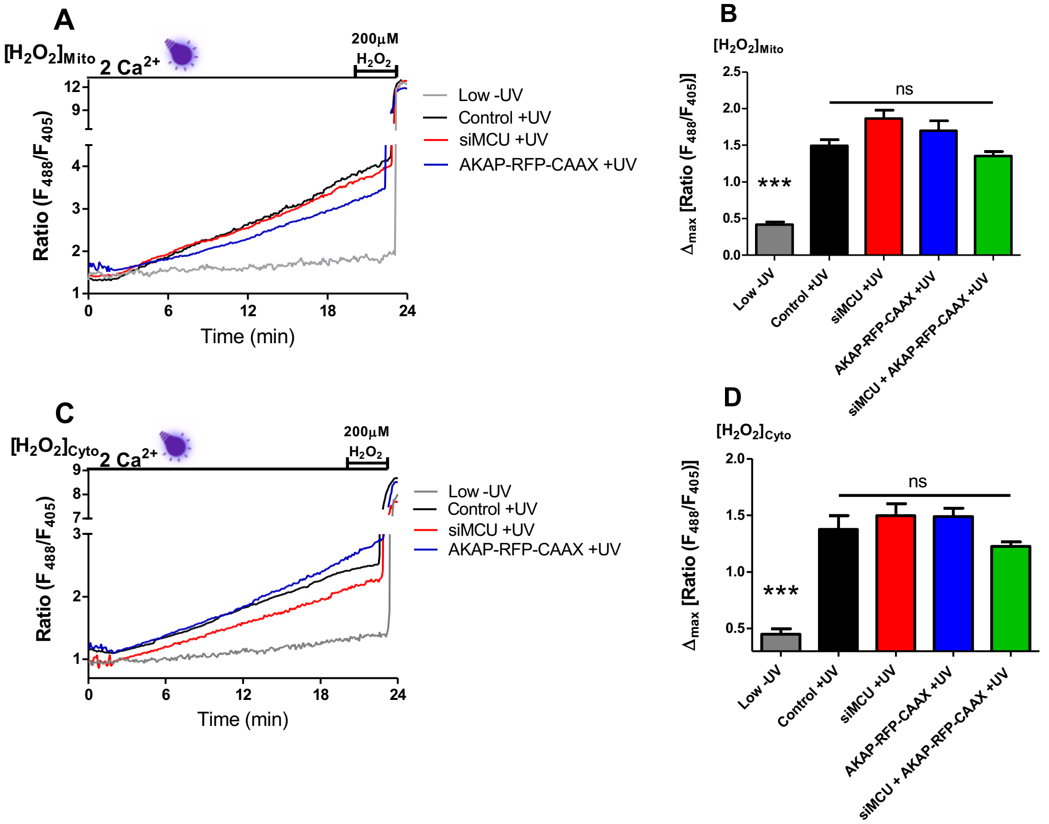

Supplementary Figure 4. High near UV light induces mitochondrial and cytosolic ROS production in siMCU, AKAP-RFP-CAAX and siMCU + AKAP-RFP-CAAX conditions. Representative traces show the production of H_2O_2 in mitochondria (A) and cytosol (C) following the excitation of cells with 5 mW of 405 nm laser line (low-UV) and 20 mW of 405 nm laser line (Control +UV, siMCU +UV and AKAP-RFP-CAAX +UV). Bar graphs represent MEAN $\pm$ SEM of H_2O_2 levels in low-UV (gray), control +UV (black), siMCU +UV (red), AKAP-RFP-CAAX +UV (blue) and siMCU + AKAP-RFP-CAAX +UV (green) in mitochondria (B) and cytosol (D). Significant differences were assessed using one-way ANOVA with Tukey's multiple comparison test and presented as specific p-values (*** $p < 0.001$, n.s. – not significant). Low-UV (n=24), Control +UV (n=46), siMCU +UV (n=19), AKAP-RFP-CAAX +UV (n=28) and siMCU + AKAP-RFP-CAAX +UV (n=13) in mitochondria and Low-UV (n=20), Control +UV (n=21), siMCU+UV (n=15), AKAP-RFP-CAAX +UV (n=16) and siMCU + AKAP-RFP-CAAX +UV (n=16) in cytosol.

A

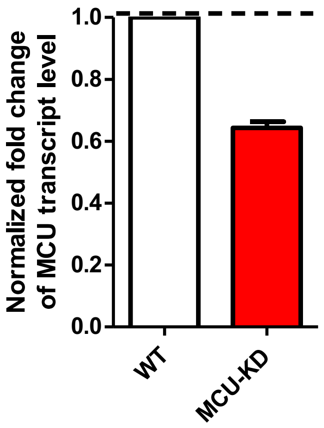

Supplementary Figure 5. Knock-down efficiency of MCU siRNA in INS-1 cells.
